# Dynamic Oxygen-Enhanced MRI of Cerebrospinal Fluid

**DOI:** 10.1371/journal.pone.0100723

**Published:** 2014-06-23

**Authors:** Taha M. Mehemed, Yasutaka Fushimi, Tomohisa Okada, Akira Yamamoto, Mitsunori Kanagaki, Aki Kido, Koji Fujimoto, Naotaka Sakashita, Kaori Togashi

**Affiliations:** 1 Department of Diagnostic Imaging and Nuclear Medicine, Kyoto University Graduate School of Medicine, Kyoto, Japan; 2 Toshiba Medical Systems Corporation, MRI Systems Development Department Otawara-shi, Tochigi, Japan; Charité University Medicine Berlin, Germany

## Abstract

Oxygen causes an increase in the longitudinal relaxation rate of tissues through its T1-shortening effect owing to its paramagnetic properties. Due to such effects, MRI has been used to study oxygen-related signal intensity changes in various body parts including cerebrospinal fluid (CSF) space. Oxygen enhancement of CSF has been mainly studied using MRI sequences with relatively longer time resolution such as FLAIR, and T1 value calculation. In this study, fifteen healthy volunteers were scanned using fast advanced spin echo MRI sequence with and without inversion recovery pulse in order to dynamically track oxygen enhancement of CSF. We also focused on the differences of oxygen enhancement at sulcal and ventricular CSF. Our results revealed that CSF signal after administration of oxygen shows rapid signal increase in both sulcal CSF and ventricular CSF on both sequences, with statistically significant predominant increase in sulcal CSF compared with ventricular CSF. CSF is traditionally thought to mainly form from the choroid plexus in the ventricles and is absorbed at the arachnoid villi, however, it is also believed that cerebral arterioles contribute to the production and absorption of CSF, and controversy remains in terms of the precise mechanism. Our results demonstrated rapid oxygen enhancement in sulcal CSF, which may suggest inhaled oxygen may diffuse into sulcal CSF space rapidly probably due to the abundance of pial arterioles on the brain sulci.

## Introduction

Oxygen causes an increase in the longitudinal relaxation rate of tissues through its T1-shortening effect owing to its paramagnetic properties, no T2-shortening effect of oxygen is seen [1], [2]. Due to such effects, magnetic resonance imaging (MRI) has been used to study oxygen-related signal intensity (SI) changes in various body organs, such as the lungs [3], brain [4], spleen, myocardium, subcutaneous fat, kidneys, bone marrow, liver and arterial blood [2], [5], [6]. The paramagnetic effect of deoxyhemoglobin has frequently been used in the brain to visualize blood-oxygen-level-dependent (BOLD) contrast as functional MRI [7], [8], and the paramagnetic effect of the oxygen molecule itself has also been used to quantify the oxygen content of cerebrospinal fluid (CSF), and to visualize oxygen enhancement (OE) of CSF on MRI [9], [10]. Fluid attenuated inversion recovery (FLAIR) imaging has been mainly used to visualize OE of CSF, since oxygen administration causes signal hyperintensity in CSF of the subarachnoid space on FLAIR [11], [12].

CSF acts as a physical cushion for the brain, and plays an important role in its biological waste disposal. This fluid is known to be produced from the choroid plexus in the ventricles, transfers to the cisterns and is eventually absorbed by the arachnoid villi, after exchanging contents with the interstitial fluid of the brain. CSF has been widely visualized using MRI techniques such as MR cisternography [13], [14], phase-contrast MRI [15], [16] and MRI with inversion pulse technique [17], which have provided clues to various pathological processes occurring in the brain. Changes in the oxygen content of CSF are reportedly associated with injury to brain tissue [18], but the resting state of oxygen content and dynamic changes in oxygen content after oxygen inhalation remain unclear. Knowledge of oxygen changes in CSF is also important from the perspective of partial volume effects during imaging analysis, since most cerebral cortices and vessels are surrounded by CSF.

OE on MR (OEMR) imaging of CSF has mostly been studied using FLAIR [1], [11], [19], T1 value calculation with inversion recovery (IR)-sequences [2], [10], and dynamic tracking has been performed with relatively longer time resolution [10], [11]. Shorter imaging time without IR will lead to better temporal resolution, but sequences without IR have not been utilized. Fast advanced spin echo (FASE) is a similar sequence with half-Fourier acquisition single-shot turbo spin-echo (HASTE), which has been previously used to calculate T1 values with IR (IR-HASTE) [2], [20]. This study compared FASE to FASE with IR (IR-FASE) in terms of the ability to dynamically track OE of CSF. We also compared OE of sulcal CSF (CSFs) with that of ventricular CSF (CSFv) in each image.

## Materials and Methods

### Subjects

The approval of the ethics committee of Kyoto University (approval number: C491) and written informed consent were obtained. Fifteen healthy volunteers (12 men, 3 women; mean age, 32±6 years) were recruited, and written informed consent was obtained from all volunteers prior to enrolment.

### MRI parameters

IR-FASE and FASE images were acquired using a 3-T MRI scanner (Toshiba Medical Systems, Otawara, Japan) using a 13-channel head coil and the following parameters in a single-slice axial acquisition at the level of 15 mm superior to the anterior commissure – posterior commissure line. **IR-FASE:** repetition time (TR), 9575 ms; echo time (TE), 48 ms; inversion time (TI), 1915 ms; matrix, 192×192, 1.37×1.37 mm; slice thickness, 7 mm; field of view (FOV), 263×263 mm; flip angle, 90°; bandwidth, 977 Hz/pixel; Number of averaging is two with additional one TR for echo stabilization. **FASE:** TR, 4500 ms; TE, 48 ms; matrix, 192×192, 1.37×1.37 mm; slice thickness, 7 mm; FOV, 263×263 mm; flip angle, 90°; bandwidth, 977 Hz/pixel; Number of averaging is two with additional one TR for echo stabilization.

### Dynamic OEMR

Using IR-FASE (28.8 s/image, 38 measurements) and FASE (13.5 s/image, 80 measurements) to track OE of CSF, images were divided into three phases: 1) pre-oxygen administration (Pre-O_2_), where subjects breathed normal room air (21% O_2_) for 5 min; 2) 100% oxygen administration, where subjects breathed 100% O_2_ at a flow rate of 15 L/min for 5 min; and 3) post-oxygen administration (Post-O_2_), where subjects breathed normal room air (21% O_2_) for 8 min. Oxygen was delivered through a non-rebreather mask that was firmly attached to cover the mouth and nose of the subject.

### Image analysis

**Figure 1:**
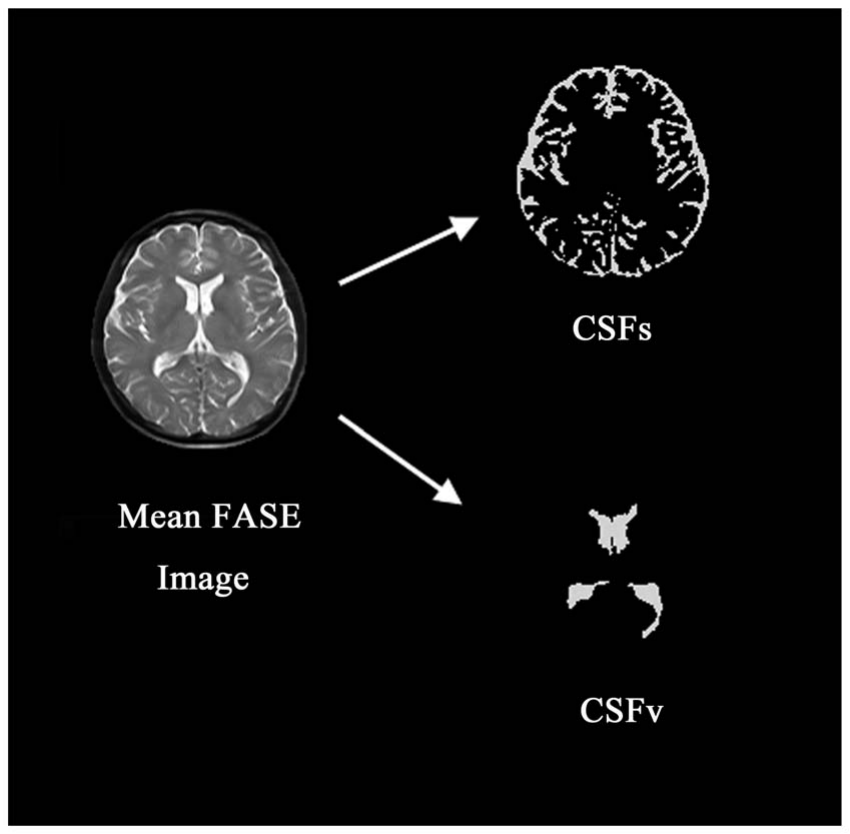
The mean image from FASE is segmented to create CSFs and CSFv masks, which are later applied to each image and total SI values for each image are calculated. The same process is performed for IR-FASE images. Note that the cavum velum interpositum was not included neirther to CSFv nor CSFs since it belongs to the cistern and it is not located at surfaces.

Images from each subject were segmented into CSF and non-CSF components using a trainable segmentation plugin of Fiji [21]. The segmented image was further processed as follows: a CSFs mask image and a CSFv mask image were created. CSFs and CSFv masks were then applied to each image (38 images for IR-FASE, 80 images for FASE) and total SI values for each image were calculated (Fig. 1).

Normalization was achieved by setting the mean SI of all dynamic images of Pre-O_2_, O_2_ and Post-O_2_ as 1000. Maximum SI (maxSI) of CSF was calculated for each subject from the start of O_2_ inhalation. Slope of SI (SI_slope_) was calculated using a differential function for SI curve and maximum SI_slope_ (maxSI_slope_) of CSF was then calculated for each subject from the start of O_2_ inhalation. Approximation methods were determined by selecting best R^2^ value for each approximation: linear curve fitting regression analysis was performed for the Pre-O_2_ and Post-O_2_ phases and polynomial curve fitting regression analysis for O_2_ phase. Mean SI with standard error of CSFs and CSFv signal values in both IR-FASE and FASE independently, and R^2^ values were calculated for each phase.

### Statistical analysis

Paired Student's t-test was conducted to compare maxSI and maxSI_slope_ values for each volunteer in CSFs and CSFv between IR-FASE and FASE, with values of *p*<0.05 considered statistically significant. MedCalc version 12.2.1 software (MedCalc Software, Mariakerke, Belgium) was used.

## Results

### IR-FASE images

**Figure 2:**
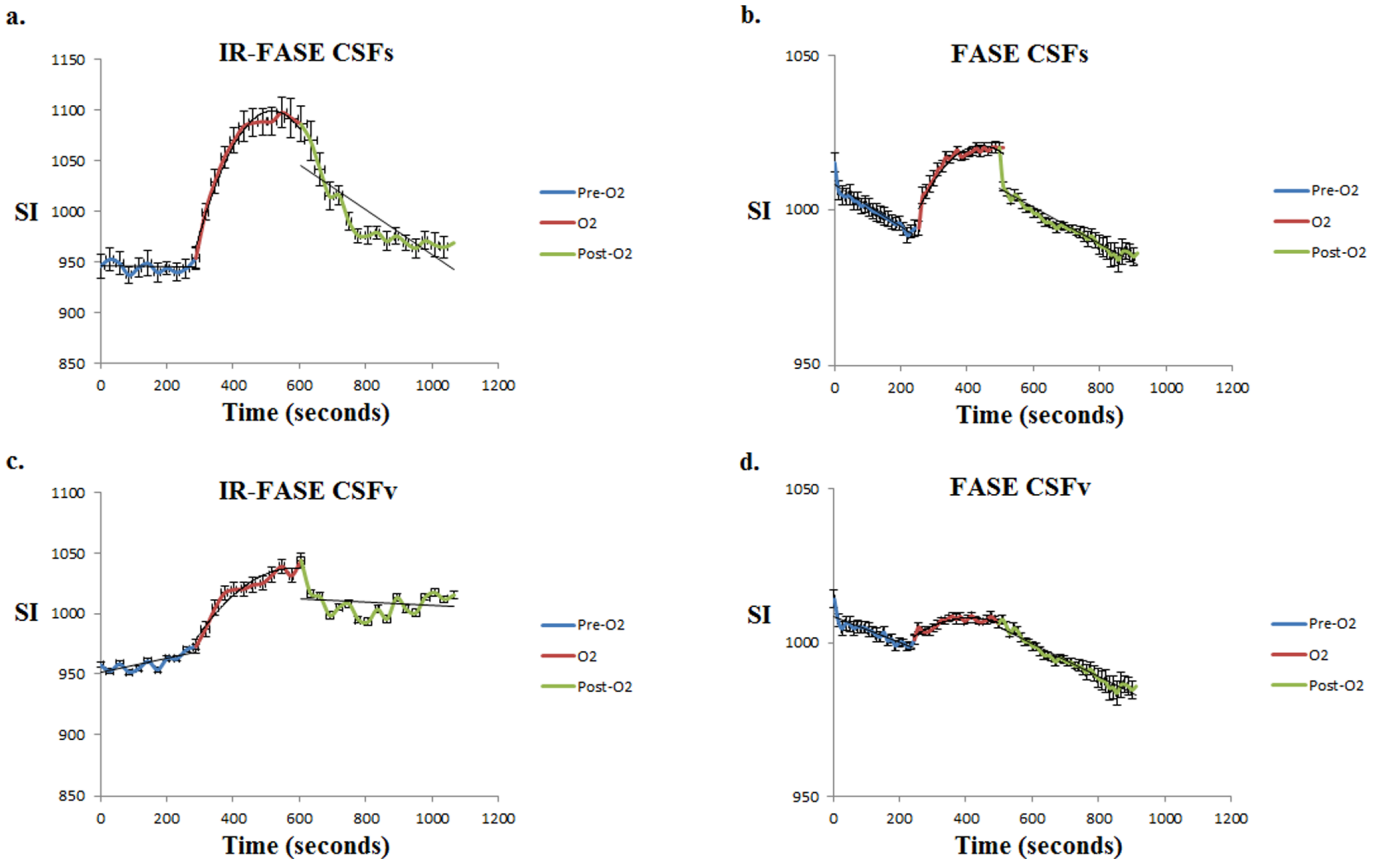
IR-FASE for Pre-O_2_, Post-O_2_ and O_2_ phases of OE-MRI of CSFs (a) and CSFv (c), show higher SI with oxygen administration in CSFs compared to CSFv. FASE for Pre-O_2_, Post-O_2_ and O_2_ phases of OE-MRI of CSFs (b) and CSFv (d), showing higher SI with oxygen administration in CSFs compared to CSFv. All data were shown with mean ± standard errors.

Pre-O_2_ showed a linear correlation with time (R^2^ = 0.06 for CSFs, and R^2^ = 0.61 for CSFv). With oxygen administration, signal values of CSF increase in correlation with time, with a better polynomial curve fit for CSFs than for CSFv (R^2^ = 0.97 for CSFs, and R^2^ = 0.76 for CSFv). Post-O_2_ signal values decrease with time, showing a linear curve fit (R^2^ = 0.68 for CSFs, and R^2^ = 0.02 for CSFv, respectively) (Fig. 2a, c).

### FASE images

Pre-O_2_ signals showed a linear correlation with time (R^2^ = 0.88 for CSFs, and R^2^ = 0.77 for CSFv). With oxygen administration, signal values of CSF rose in correlation with time, with a better polynomial curve fit in CSFs than CSFv (R^2^ = 0.94 for CSFs, R^2^ = 0.72 for CSFv). Post-O_2_ signal values decrease with time, showing a linear curve fit (R^2^ = 0.94 for CSFs, and R^2^ = 0.95 for CSFv) (Fig. 2b, d).

### Subtraction images

**Figure 3:**
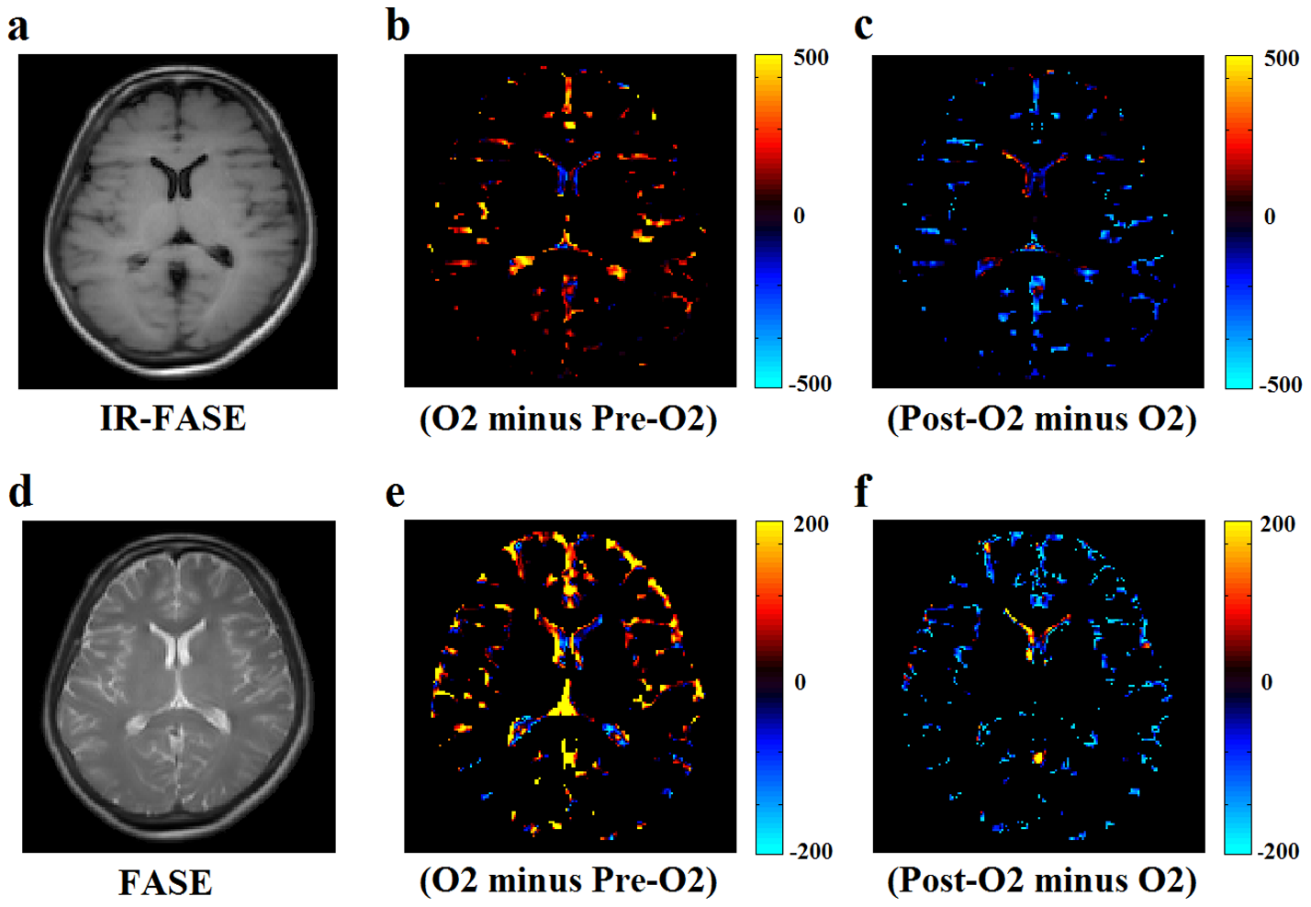
IR-FASE image (a), calculated IR-FASE images: “O_2_ minus Pre-O_2_” (b), “Post-O_2_ minus O_2_” (c). FASE image (d), calculated FASE images: “O_2_ minus Pre-O_2_” (e), “Post-O_2_ minus O_2_” (f). Calculated images of “O_2_ minus Pre-O_2_” show a positive SI difference (b, e), while calculated images of “Post-O_2_ minus O_2_” show a negative SI difference (c, f). Intra-ventricular high signals in “Post-O_2_ minus O_2_” images are assumed to come from the highly vascular choroid plexus.

O_2_ minus Pre-O_2_ shows a positive SI difference, while Post-O_2_ minus O_2_ shows a negative SI difference in both IR-FASE and FASE images (Fig. 3).

### IR-FASE vs. FASE

#### CSFs

**Figure 4:**
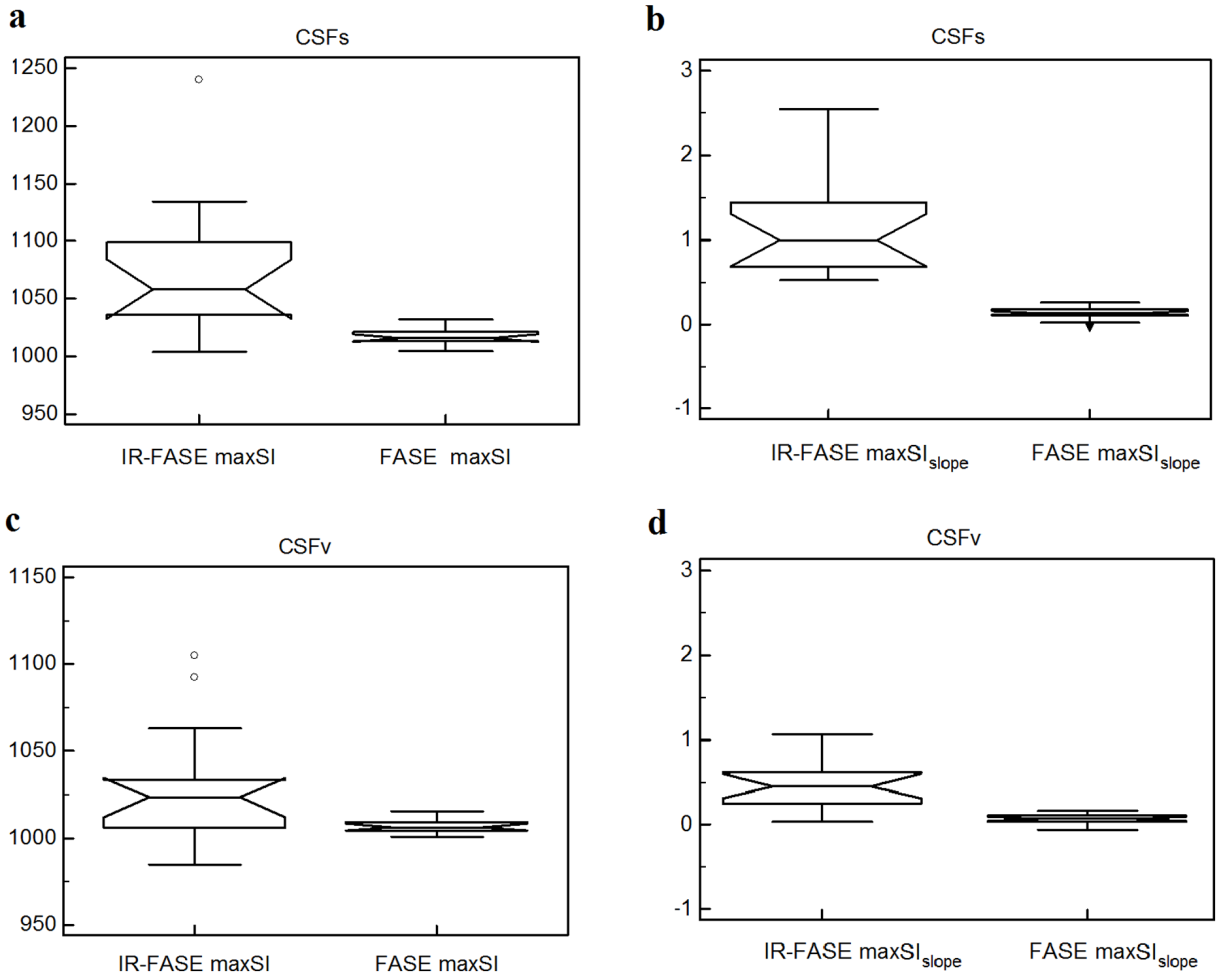
Box-and-whisker plots of IR-FASE vs. FASE maxSI and maxSI_slope_ of CSFs (a–b) and CSFv (c–d). **CSFs**: maxSI and maxSI_slope_ are significantly higher for IR-FASE than for FASE (p = 0.001 and p<0.0001, respectively) (a, b). **CSFv**: maxSI and maxSI_slope_ are significantly higher for IR-FASE than for FASE (p = 0.034, and p = 0.0001 respectively) (c, d).

Values of maxSI and maxSI_slope_ were significantly higher for IR-FASE than for FASE (p = 0.001 and p<0.0001, respectively) (Table 1, Fig. 4a–b).

**Table 1:** MaxSI and maxSI_slope_ for IR-FASE and FASE.

	maxSI			maxSI_slope_		
	IR-FASE	FASE	IR-FASE vs. FASE	IR-FASE	FASE	IR-FASE vs. FASE
CSFs	1073.1±52.8	1015.9±8.3	P = 0.001*	1.277±0.639	0.197±0.078	P<0.0001*
CSFv	1028.1±34.3	1008.1±6.5	P = 0.034*	0.463±0.287	0.061±0.06	P = 0.0001*
CSFs vs. CSFv	P = 0.004*	P<0.0001*		P = 0.0003*	P<0.0001*	

All data were shown with mean ± standard errors. *Statistical significance (P<0.05).

#### CSFv

Values of maxSI and maxSI_slope_ were significantly higher for IR-FASE than for FASE (p = 0.034, and p = 0.0001, respectively) (Table 1, Fig. 4c–d).

### CSFs vs. CSFv

**Figure 5:**
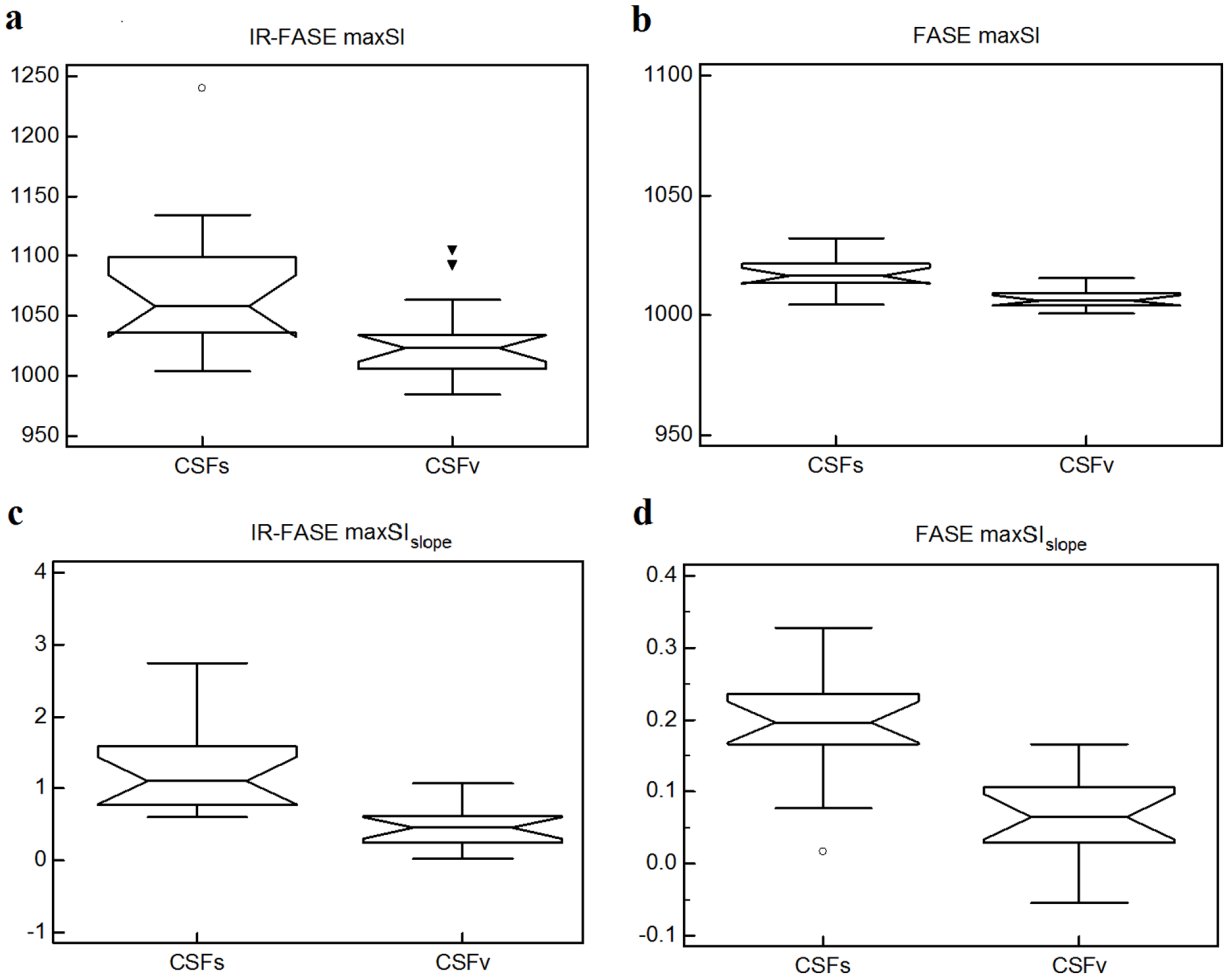
Box-and-whisker plots of CSFs vs. CSFv. Values of maxSI and maxSI_slope_ are significantly higher for CSFs than for CSFv in both IR-FASE (maxSI, p = 0.004; maxSI_slope_, p = 0.0003) (a, c) and in FASE (maxSI, p<0.0001; maxSI_slope_, p<0.0001) (b, d).

CSFs showed significant higher maxSI and maxSI_slope_ than CSFv in both IR-FASE (p = 0.004 and p = 0.0003 for maxSI and maxSI_slope_, respectively) (Table 1, Fig. 5a–b) and FASE (p<0.0001 and p<0.0001 for maxSI and maxSI_slope_, respectively) (Table 1, Fig. 5c–d).

## Discussion

This study demonstrated dynamic tracking OE of CSF on both IR-FASE and FASE, since both methods showed positive signal increases during O_2_ administration and maxSI data supported these findings. Rapid OE after O_2_ administration displayed by SI_slope_ was also demonstrated and represented by maxSI_slope_. In addition, OE of CSFs was visualized better than OE of CSFv on both IR-FASE and FASE. OE differences between CSFs and CSFv appeared largely compatible with previous studies of FLAIR with 5-min imaging sequence [1] and T1 value calculation with 7-min imaging sequence [10], particularly in terms of the regional differences of T1 in CSF spaces such as basilar cisterns, lateral ventricles and cortical sulci. IR-FASE showed more OE of CSFs than FASE. Our results support previous reports of differences between OE of CSFs and OE of CSFv, but with higher temporal resolution than previously described [1], [10].

CSF is traditionally thought to mainly form from the choroid plexus in the ventricles and is absorbed at the arachnoid villi [18], [22]. While it is also believed that cerebral arterioles contribute to the production and absorption of CSF [23], controversy remains in terms of the precise mechanisms [24]. Capillaries in direct contact with the CSF form the blood-CSF barrier, where many constituents pass from the intra-arterial environment into CSF [25]–[27]. Since a linear relationship exists between arterial partial oxygen pressure (PaO_2_) and CSF oxygen tension, increasing PaO_2_ levels with 100% O_2_ administration will lead to increased CSF levels of O_2_. Oxygen diffuses into the CSF through the blood-CSF barrier, and this exchange occurs more in CSFs than in CSFv, due to the abundance of pial vessels on the surface of the brain compared to intra-ventricular vessels. The larger amount of intra-ventricular CSF might also cause more dilution of oxygen. Another cause might be the leaky areas of the blood-brain barrier near the pituitary gland, which would further facilitate oxygen diffusion into CSFs more than into CSFv. All of these mechanisms might contribute to the difference in OE between CSFs and CSFv [1]. A close relationship exists between CSF and arterial flows. Phase-contrast cine MRI has revealed the age-dependence of CSF flow increases and correlations with arterial flow [28]. Our results partly supported the idea that cerebral vessels are engaged in CSF production, since oxygen rapidly transfers to CSF from arteries.

IR sequences on MRI have been used to study OE of CSF in most previous reports [1], [11], [12]. Such OE was noticed on FLAIR images of anaesthetized patients and was initially explained by the effects of propofol, which has a similar T1 to CSF that would lead to incomplete nulling of the CSF signal [19]. However, later studies interpreted the hyperintensity as due to the paramagnetic effects of oxygen on CSF, in turn causing T1-shortening effects [11], [12], [29]. Arterial oxygen saturation by hemoglobin is close to 100% in healthy individuals. The partial pressure of dissolved oxygen in the blood represents a small fraction of the total oxygen concentration (less than 0.3%). The concentration of dissolved oxygen in the blood increased after oxygen inhalation and dissolved oxygen in the blood will diffuse into tissues according to the oxygen pressure gradient [1]. FLAIR is the sequence with an IR pulse to nullify the signal of CSF, so changes in T1 relaxation time of CSF interfere with suppression of the CSF signal. The T1-shortening effect has been utilized on FLAIR in various situations [30]. As HASTE sequence was used to calculate T1 values with IR [2], IR-FASE provides a similar T1-shortening effect induced by oxygen as FLAIR, so FASE without IR might also show a weak T1-shortening effect. Since FASE shows T2 contrast, a T2-elongation effect cannot be excluded, although previous reports have claimed no apparent change in T2 relaxation time with oxygen administration [2]. The precise mechanisms remain unclear, and this appears to be the first study to dynamically track OE of CSF on FASE images.

OE-MRI might act as a non-invasive method for delineating pathological lesions occupying CSF spaces [31], and to better understand changes in oxygen status in the case of CSF-filled cystic tumors. OE-MRI might have the potential to non-invasively visualize cerebral collateral blood supply in cases of carotid occlusive disease through the diffusion of oxygen into CSF.

Several limitations exist, since these methods require a high degree of compliance from the imaged subject; firm fixation of the head was conducted in this study to reduce minor motions of the head. Second, quantification of OE such as T1 value calculation was not conducted in this study. We focused on higher temporal resolution of OE in this study, but rapid T1 calculation imaging is expected in the near future.

In conclusion, rapid oxygen enhancement of CSF can be dynamically tracked with both IR-FASE and FASE, and is observed more in CSFs than in CSFv, probably due to the abundance of pial arterioles on the brain surface compared to the intra-ventricular arterial system.
